# Action-Depicting Gestures and Morphosyntax: The Function of Gesture-Speech Alignment in the Conversational Turn

**DOI:** 10.3389/fpsyg.2021.689292

**Published:** 2021-07-30

**Authors:** Paweł Urbanik, Jan Svennevig

**Affiliations:** ^1^Center for Multilingualism in Society Across the Lifespan, Department of Linguistics and Scandinavian Studies, University of Oslo, Oslo, Norway; ^2^Department of Nordic and Media Studies, University of Agder, Kristiansand, Norway

**Keywords:** action depiction, stroke position, gesture-syntax alignment, projection, verbal affiliate, multilingual construction site, interaction, gestures

## Abstract

The current study examines the role of action-depicting gestures in conversational turns by focusing on their semantic characteristics and temporal position in relation to their verbal affiliates (action verbs or more complex verb phrases). The data are video recordings of naturally occurring interactions in multilingual construction sites in Norway. The analysis distinguishes two modes of action depiction: generic depictions, which represent the action as a general type, and contextualized depictions, which in addition include deictic references to the spatio-material environment or iconic representations of the specific manner of action performance. These two modes typically occupy different positions in the turn. Generic depictions are mostly initiated before the verbalization of the action or are synchronized with it, while contextualized depictions mostly start simultaneously with the verbalization and extend beyond the verb phrase or the turn. The pre-positioned and synchronized generic gestures are shown to serve as a practice for facilitating recognition of the verbalized action and may be temporally manipulated in order to pre-empt understanding problems in the face of reduced common linguistic resources. The post-positioned contextualized depictions serve instead to add specifying information about aspects of the action referred to and thereby to complement or supplement the meaning of the verb phrase, securing understanding of action specifics. The study contributes to research on gesture-speech synchrony by demonstrating how variation in the alignment of action depiction and syntax is used to direct the recipient’s attention toward different interactional goals.

## Introduction

The concept of *action* is relevant for analyzing conversation in two fundamentally different ways. First, conversational contributions are performative (Austin, 1962) in the sense that they accomplish *social actions* (Schegloff, 1986) or communicative acts (Clark, 1996) vis-à-vis the recipient(s). The interactional process of action formation (and ascription) is based on the contribution’s composition (its reliance on conventional linguistic and embodied resources) and position in a sequence of turns (mainly its status as a response to the previous action) in a given contextual configuration (cf. Enfield and Sidnell, 2017). Second, conversational contributions often *refer* to actions as part of their semantic or ‘ideational’ meaning. Such actions are typically conveyed by action verbs or more complex verb phrases. They may concern actions in the here-and-now situation or displaced actions, performed by the speaker or by some other actor. In addition to the verbal expression, they may also be represented by gestures depicting aspects of the performance. In Extract 1, we illustrate how these two forms of action are relevant to analyzing a conversational contribution by presenting a short sequence from a meeting in which a construction worker, Tomasz, proposes a solution to his foreman, Georg, on how to secure a construction so that a task can be performed.

**EXTRACT 1:**
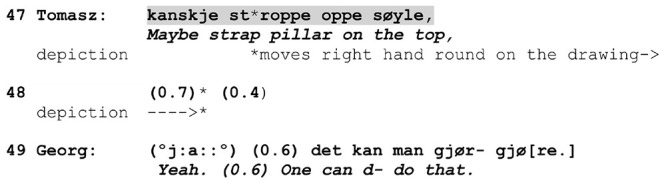
L2 Norwegian.

In this excerpt, Tomasz performs the social action of making a *proposal* to Georg about how to proceed in a work task. The social action exploits a conventional action format for proposals, namely the adverb ‘maybe’ plus a verb in the infinitive, and it comes at a point in the conversation where making a suggestion is expectable, as the workers have been talking about potential solutions. In making the proposal, Tomasz refers to the physical action of ‘strapping’ something. In addition to using the action verb *stroppe* (‘strap’), he represents the action by performing a gesture in which he moves his hand round with the index finger pointing at the construction drawing, thereby depicting the manner of action performance. The depiction begins during the verbalization of the action and extends beyond the turn.

In this article, we will focus on how actions named in speech are represented by a combination of verbal and gestural means, so-called *action-depicting gestures.* More specifically, we investigate semantic and temporal relations between the gestures and their verbal affiliates. We address two questions: 1) What is the specific contribution of action-depicting gestures to the representation of physical actions in conversational turns? 2) How do speakers position their gestures in relation to the verbal affiliates and what interactional consequences does this have? In answering these questions, we will also take into account the relevance of the first (social) concept of action representation to the emergence of action-depicting gestures in the sequential unfolding of talk.

The relationship between gesture and speech has been studied for a long time and from different theoretical and methodological perspectives. Regardless of the research field, the predominant view is that gestures are integral to human language systems and that they are co-expressive with speech, meaning that both, although very different, modes of expression work together to form meaningful conversational units (Clark, 1996; Kendon, 2000; McNeill, 2005; de Ruiter, 2007; Enfield, 2009). However, insight into the details of this composition is naturally a matter of how the speech-gesture orchestration is approached. In the psycholinguistic tradition, the focus is on the processual aspects in the human mind and different views concern the link between gesture and thought and the cognitive mechanisms of gesture production. More specifically, the dispute is about the representational source of gesture generation and the extent to which gesture is integrated with speech during the production process (de Ruiter, 2000, 2007; Krauss et al., 2000; Beattie, 2003; Goldin-Meadow, 2003; Kita and Özyürek, 2003; McNeill, 2005; Hostetter and Alibali, 2008). In the interaction-oriented tradition, researchers pay closer attention to the role of gestures in daily encounters. Gestures are considered to co-participate in the process of meaning making by providing semantic content that may be either complementary (i.e., additional to what is conveyed in speech) or supplementary (i.e., not conveyed in speech). In addition, they also serve as resources to organize social interaction. They arrange it spatially and temporally by constructing spaces of mutual orientation, changing them in time, and signaling prominence in speech (Goodwin, 1986; Kendon, 1995; Streeck, 2009a). They provide information about the on-going activity and are consequential for the actions and events that constitute it (Goodwin and Goodwin, 1986; Gullberg, 1998; Kendon, 2004). Participants use gestures to compose, perform, prompt or complete various social actions and secure their recognizability (Kendon, 1995; Olsher, 2004; Sidnell, 2006; Goodwin, 2018; Lilja and Piirainen-Marsh, 2019). Gestures can also foreshadow verbalizations, actions and stances (Schegloff, 1984; Streeck and Hartge, 1992; Streeck, 2009b; Lilja and Piirainen-Marsh, 2019) and facilitate language processing, accelerating the progressivity of conversational activities (Holler et al., 2018). This tradition of gesture research has shown how gesture and speech “mutually elaborate and constrain each other” (Goodwin, 2018:336) and how they, by interaction with other modalities and with the material world (Goodwin, 2007, 2018; Streeck, 2009a), propel the socio-cognitive machinery, guiding the intersubjective cooperation between interlocutors and the sequential organization of their interaction.

However, what is still underexplored is the interactional mechanisms of gesture production, including the local significance of its temporal relation to verbalized structures. This study fills this gap by exploring the speech-gesture combination in authentic interactions from construction sites. In particular, we investigate gestures’ semantic, positional and interactional properties by looking at how action depiction relates to action verbalization at the level of syntax and of interaction.

### Action Depiction

Among many ways in which people employ gesture is the one that consists in depicting the properties of actions or objects by using arms, hands and fingers. However, despite a long-lasting interest in gestures, depiction on its own has received surprisingly little attention. In general reviews, depictive gestures have been treated as parts of illustrators (Ekman and Friesen, 1969), imagistic gestures and their subclass – iconics (McNeill, 1992), or representational gestures in general (Kendon, 2004). Some researchers additionally distinguish between object depiction and action depiction. Ekman and Friesen (1969) introduce pictographs that illustrate referents and kinetographs that represent bodily actions. Kendon (2004) makes a distinction between modeling (shaping a form that represents an object), enactment (illustrating a pattern of action), and depiction (sketching a virtual object), while Streeck (2008) identifies a number of different modes, such as modeling, drawing, handling (object represented through an action), and acting or mimesis (representation of a practical action).

Recently, depiction has been approached in a more focused manner from two perspectives. Streeck (2008, 2009a) sees it as an action that draws on the experiential knowledge of our everyday manual acting. Thus, depictive gestures reflect how we interpret actions and things based on what we already know about them and their applications. This corresponds with recent evidence from psycholinguistic research showing that experience with an action facilitates comprehension of its language representation and helps to discriminate between actions (Goldin-Meadow and Beilock, 2010; Cartmill et al., 2012). The second perspective is proposed by Clark (2016) who describes depictions as “physical analogs” staged as scenes representing other scenes. Their mechanisms follow principles that take into account several factors, such as the role of the depictor in the depiction (cf. McNeill, 1992 on gesture viewpoint), the depiction’s elements, and spatial and temporal frames of reference. Action depiction in both approaches is mentioned as one of many ways of representing events, things and behavior, but it has not received any systematic analysis.

More detailed investigations have recently come from Conversation Analysis and Interactional Linguistics. Keevallik (2013) has studied how syntactic elements work together with bodily demonstrations in dance instructions, composing combined units of language expression. Her analysis provides insight into the constraining and projective role of syntax and the information richness of embodiment. Lilja and Piirainen-Marsh (2019) have, in turn, looked at the role of depictive gestures produced during the final components of conversational turns. They show that action depiction serves to elaborate and specify the verbal component and thus facilitate the recognition of social action, projecting next actions and sustaining the progressivity of interaction. These studies also confirm previous observations about representational gestures with regard to their selective character (i.e., aspects of action they illustrate), the guiding role of speech in their interpretation, and their specifying contribution to speech content (cf. Kendon, 1995, 2004; Streeck, 2009a).

### Gesture Position

Gestures occur at given points of interactional units, being temporally coordinated with speech in various ways. Investigation of gesture-speech (a)synchrony, crucial for many cognitive models of gesture generation, has a long tradition and reveals some disagreements and challenges. These mainly concern the methods of measuring the temporal relation between gesture and its lexical affiliate (a verbalization that semantically corresponds most closely to the gestural representation; Schegloff, 1984) but also the identification of the latter. Some researchers consider the beginning of the preparation phase as the point of departure while others – the moment of stroke onset. Consequently, the reported results differ. It is widely accepted that the onset of a whole gesture phrase most often comes before the lexical affiliate (Schegloff, 1984; Morrel-Samuels and Krauss, 1992; Hadar and Butterworth, 1997; McNeill, 2005; Church et al., 2014; ter Bekke et al., 2020) and occurs during speech not pauses (Nobe, 2000; Chui, 2005), but there are inconsistent findings concerning the gesture stroke. Several studies of various languages have demonstrated that the stroke onset starts (and sometimes even ends) before the affiliate (Schegloff, 1984; Ferré, 2010; Bergmann et al., 2011; ter Bekke et al., 2020) while several others report that it is synchronized with the affiliate (Chui, 2005; Graziano et al., 2020) or the co-expressed speech (McNeill, 2005), which automatically implies that the gesture onset is prepositioned anyway. What many of these studies additionally show is that strokes produced after the affiliate turn out to be rare.

Prepositioned gestures are claimed to be predominant due to faster access to motoric representations (Wagner et al., 2014). Their earlier production is considered to bear predictive potential (Schegloff, 1984; ter Bekke et al., 2020) and strengthen the perceived focus in speech (Treffner et al., 2008). The temporal asynchrony is also explained by means of semantic closeness between the gesture and the speech content, showing that semantic familiarity and similarity decrease asynchrony (Morrel-Samuels and Krauss, 1992; Bergmann et al., 2011). However, the identification of a lexical affiliate is not always an easy task because gestures may include information verbalized with several lexical components while researchers most often analyze gestures in aggregated categories (e.g., iconic or representational) without being clear about how they approach these challenges or paying attention to the role of the elements that are not co-expressed with gestures. Another issue is a single-point focus on gesture production (its onset) that marginalizes the temporal span of gesticulation and its alignment with the syntactic structures of speech. In other words, apart from being employed in relation to certain lexical components, gestures also appear at given points of syntactic units and interactional turns and vary in time. A growing body of research shows that the structural positioning of gestures is equally important as their temporal positioning. Gestures are used to substitute or fulfill certain syntactic constructions (Olsher, 2004) and they accompany certain words, phrases or clauses more often than others (Kok, 2017; Debreslioska and Gullberg, 2020). At the level of turn-taking, their application serves concrete interactional goals, such as predicting turn ends and the next turns or actions (Streeck, 2009b; Lilja and Piirainen-Marsh, 2019), which requires manipulation of gesture timing (e.g., stroke prolongation). This indicates that variation in the temporal management of gestures plays a role in organizing interaction. However, the issue that has not yet received attention is how this variation is realized in naturally occurring interaction. The gesture-speech (a)synchrony has not been systematically analyzed with regard to its alignment with syntactic units and interactional turns. More specifically, there is little knowledge of how the semantic relation between gesture and its affiliate is functionally linked with the syntactic matrix and its sequential position. A closer look at this configuration allows us to find out what it means that a stroke depicting an action in one way or another starts and ends at a given point of an interactional turn. This is exactly what this study aims to achieve.

### The Current Study

The present article adds to research on gestures in general and on depiction in particular by narrowing the study object to action-depicting gestures and systematically analyzing their occurrence in talk. Specifically, we look at how construction-site workers illustrate actions they are referring to in speech and how they manage their gestures temporally. The choice of construction-site settings offers a significantly different environment than the ones that are used in experimental and interactional studies focusing on gesture-speech synchronization. Utterances conveying that something is done or how it can or should be done are particularly important in construction-sites, as they organize work activities and affect their progression. As a result, workers make much use of action verbs accompanied by action-depicting gestures in order to describe, identify or evoke physical actions and activities. Furthermore, their interactions occur in surroundings whose elements are often directly relevant to the on-going talk and must be oriented to. This adds to the analysis an aspect of spatio-material environment and its role in action depiction, providing new insights into the temporal alignment of gestures with speech in less explored contextual configurations.

In addition, the construction sites are highly multilingual environments, with workers from mainly Poland, Sweden and Norway speaking Norwegian as a lingua franca. Some of the workers, among them our main participant, Tomasz, have limited proficiency in the language, and thus tend to rely on gestures to complement their often unidiomatic or rudimentary verbal utterances.

The study has two goals that serve to identify the interactional mechanisms of temporal orchestration of gestures and speech. First, it seeks to characterize action depiction in terms of its semantic relationship with speech affiliates in its natural conversational environment and various contextual configurations. Second, it aims at investigating the interactional grounds and implications of the positional variation of action-depicting gestures relative to their affiliates.

## Materials and Methods

### Data

The data consist of approximately 6 h of video recordings of interactions between a Polish worker (‘Tomasz’) and his co-workers and superiors at two construction sites in Norway. The recordings were made by the worker in question (on-site footage) and by two researchers (in-office footage). They document interactions during work tasks where Tomasz directs other workers’ actions or consults on tasks with the co-workers and superiors and during conversations in which the participants plan the performance of various tasks. The on-site recordings were made with a camera mounted on Tomasz’s helmet. This has some limitations for the visibility of some of his gestures. Therefore, only gestures with a clearly visible stroke phase were taken into account.

In order to identify action-depicting gestures, we focused on arm-hand-finger movements that represented aspects of the actions named in speech. Particularly, we focused on the stroke phase, from its onset to the onset of post-stroke hold or retraction, as this was the critical and least ambiguous moment in which the gesture conveyed the most crucial information about action (Schegloff, 1984; McNeill, 1992, 2005; Kendon, 2004). If a gesture consisted of multiple strokes, we delimited all of them if this was possible but marked the whole time span of their occurrence as one unit of action depiction. Consequently, a stroke phase in our study may include more than one stroke.

In total, we have identified 92 gestures that illustrate actions co-expressed in speech with verbal affiliates, i.e., action verbs (e.g., *spikre* ‘to nail’) or larger verb phrases (e.g., *ha varme på*, ‘to run the heating’) that name the action in question.

### Participants

**FIGURE 1:**
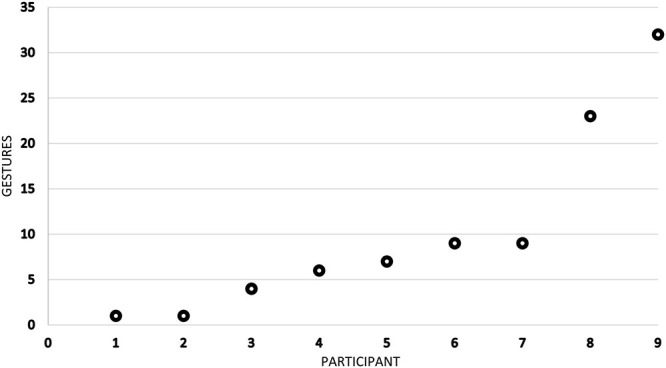
Frequency distribution of action-depicting gestures in the data (individual values sorted from smallest to largest). Mean = 10.22, SD = 10.47, *n* = 9.

The 92 action-depicting gestures were produced by 9 male participants, young and middle-aged adults. 3 of them speak Norwegian, 4 of them speak Swedish or a mixture of Swedish and Norwegian (the so-called *svorsk*) while 2 of them speak L2 Norwegian.^1^ Among these L2 Norwegian speakers is Tomasz, who also communicates in Polish with his native co-workers. Since the recordings concentrate on his workplace interactions, the distribution of the identified gestures is skewed, as almost three-fifths of all depictions are produced by Tomasz (no. 9 in Figure 1) and one of his Swedish superiors whom he frequently consults (no. 8 in Figure 1).

The data have been collected by permission of the Norwegian Center for Research Data (NSD). The participants received oral and written information about the project before signing their consent to participation. They also gave consent for the video recordings and photos to be used for academic dissemination in anonymized form.

### Procedure

Talk and gestures were annotated in ELAN (2020). Gesture annotation was preceded by a frame-by-frame analysis and verified twice. After the sequential analysis of each conversational instance, the identified gestures were additionally coded according to the name of participant, the language of co-expression, workplace settings, stroke position relative to the verbal affiliate, the number of strokes, and the temporal length of stroke phase (measured in milliseconds). Each gesture was also described in terms of motoric characteristics, its semantic content, its position in the sequential organization of interaction (i.e., relative to the preceding and the following turn), and the social action it accompanied. In general, action-depicting gestures in our data are mostly one-stroke enactments (64%) and they most often occur in various first actions (often instructions, directives, and explanations, but also proposals or questions for confirmation, among others). They accompany a large variety of different action verbs (49 lexemes) some of which (e.g., ‘lay’, ‘come’, ‘put’) are more commonly used than others. Table 1 presents an overview of the features that are relevant for the current study.

**TABLE 1:** Dstribution of action-depicting gestures according to the language of co-expression, the settings of production, and the position of stroke in relation to the verbal affiliate.

**Language of co-expression**	***n* (% of 92)**	**Workplace settings**	***n* (%of 92)**	**Stroke position**	***n* (% of 92)**
Swedish or Swedish-Norwegian	**40 (43.5%)**	Site	**54 (58.7%)**	Pre-verbal	**39 (42.4%)**
L2 Norwegian	**29 (31.5%)**			Post-verbal	**31 (33.7%)**
Norwegian	**14 (15.2%)**	Office	**38 (41.3%)**	Verb-synchronized	**14 (15.2%)**
Polish	**9 (9.8%)**			Cross-extending	**8 (8.7%)**

**FIGURE 2:**
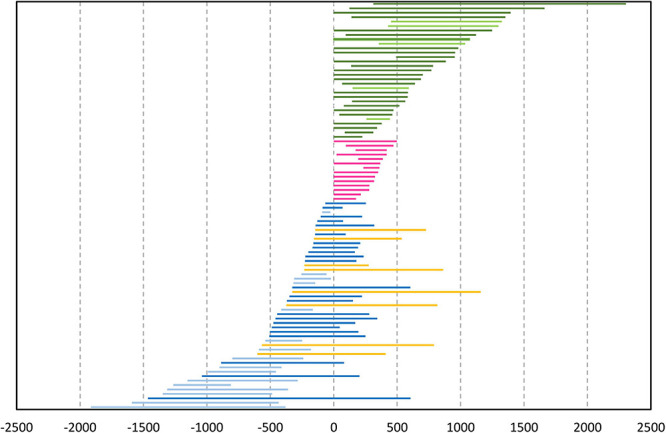
Position and duration of stroke phases (*N* = 92) in relation to their verbal affiliates in the data (measured in milliseconds; 0 marks the onset of the affiliate). Yellow lines mark gestures with cross-extending strokes, while pink lines mark verb-synchronized gestures. Lighter tones in the color categories denote gestures that end before (blue) or begin after (green) the verbalization of the affiliate.

The position and the temporal length of the stroke phase were measured and coded in relation to the onset of the verbal affiliate. In this way, we have identified four main positional categories: 1) depictions with strokes beginning before the affiliate onset and ending before or during the verbalization of the affiliate (pre-verbal gestures); 2) depictions beginning during or after the verbalization of the affiliate and extending over the rest of the talk or beyond it (post-verbal gestures), as in Extract 1; 3) depictions beginning and ending during the verbalization of the affiliate (verb-synchronized gestures); and 4) depictions with strokes beginning before the affiliate onset and expanding beyond it (cross-extending gestures). Figure 2 presents the temporal span of the identified gestures in each category.

Each stroke phase was additionally marked on the syntactic units of the co-expressed talk in order to find out at which point of speech production a given depiction started and ended. Furthermore, each syntactic unit co-occurring with an action-depicting gesture was characterized with regard to the types of components used (i.e., complements and adjuncts) and their structural position in this unit. The semantic content of each depiction was described in terms of what the gesture represented (e.g., laying something on something) relative to the verbal affiliate (e.g., the verb ‘lay’) and what additional elements it incorporated (e.g., referential cues).

The study combines Interactional Linguistics with multimodal Conversation Analysis as a method. The approach examines linguistic and embodied resources used in interaction by paying special attention to their role in building meaningful structures in the sequential organization of talk. We particularly focus on the interactional relevance of action-depicting gestures applied in different positions within syntactic units of speech. The transcripts follow conversation-analytic conventions (Jefferson, 2004) and multimodal conventions developed by Mondada (2016) complemented by morpheme-by-morpheme glosses. The exact moment of the stroke onset is marked with the letter *s* while its time span is delimited with ^∗^ (For more information on conventions, see Appendix). Under each stroke, three still images present its unfolding in time.

## Results

### Generic and Contextualized Depictions

We have identified two distinct ways in which the participants depict actions by using arm-hand-finger gestures. The first represents the action in generic terms, that is, without any specifications related to the spatio-material setting. For instance, in one example raising an arm depicted the action of lifting something, without displaying any context-specific features such as where the lifting occurred, which objects were involved or how the lifting was performed. We call these non-specifying gestures *generic depictions*. They make up 52% (n=48) of all gestures in the data (mean of individual proportions=0.52, median=0.52, SD=0.3, n=9). The second way of depicting actions consists in incorporating and displaying their context-specific features, such as deictic references to specific locations in the surroundings or a particular manner of action performance. In another example, an arm moved forward and downward depicted the action of moving something in a certain direction relative to the surroundings and laying it in a certain place. So, in addition to depicting a type of action, such gestures were enriched with additional information specifying aspects of action performance (cf. Kendon, 2004; Gerwing and Allison, 2009;:104, 185). They make up 48% (n=44) of the gestures in the data and we call them *contextualized depictions*. These specifying gestures normally rely on elements in the spatio-material configuration (cf. Goodwin, 2018). However, speakers may also exploit proxy referents established in the gesture space (see Streeck, 2009a:124 on blending of spaces), especially when the actual referents are displaced, that is, located outside the speakers’ material environment (e.g., referring to the construction site while talking in the office). As an example, one hand may represent a particular object upon which the other hand moves, representing the action of putting one object onto the other.

These two modes of action depiction manifest two different speaker orientations to what is relevant in a given situation – the action itself or its context-specific features. We exemplify both ways of action depiction in Extract 2, where Tomasz explains to Ivar (a Swedish superior speaking Swedish-Norwegian) that he has “cleaned up” the area by moving some reinforcing rods to a different place. He responds to a question from Ivar in which the latter notices that the rods have been moved from the place he refers to with a pointing gesture (line 32).

**EXTRACT 2:**
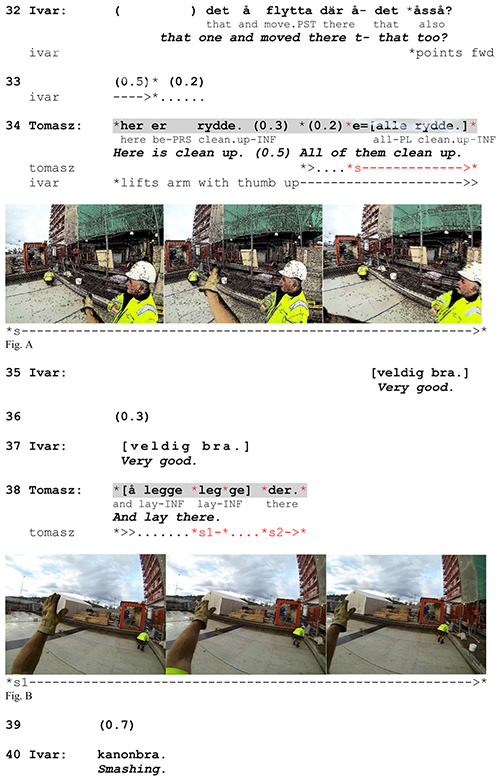
L2 Norwegian, site.

While informing Ivar that he has removed the rods, Tomasz depicts the action with a single horizontal sweeping arm movement from right to left (Figure A). This is a conventional gesture the workers use when illustrating the action of removing things from a place. Here the enactment does not provide any additional information about the action. It neither refers to any object or place in the material context (the area referred to by Ivar is not where they are standing) nor indicates the direction of the removal or the manner of execution. It generically depicts the action of ‘cleaning up’, introducing it as an action *type*. By doing so, Tomasz makes it visible to Ivar so that the latter is not only informed about it but also potentially witnesses it. The ‘witnessability’ of a gesture has been described as a way of providing evidence for an action (Nevile, 2007). In our example, this action is crucial in terms of what Tomasz aims to highlight as interactionally relevant. Importantly, he does not directly answer Ivar’s question that already includes noticing that the rods are gone but emphasizes that he has cleaned up the whole place. In other words, Tomasz provides a transformative answer (Stivers and Hayashi, 2010) in which he shifts the focus of the question to a different type of action (cleaning up the place instead of merely moving the rods). When he introduces the cleaning up, it is not the contextual aspects that are relevant but the action itself, the fact that the action has been carried out. Therefore, his gesture relies on a default and generic representation of the type of action in question. In this way, Tomasz can facilitate and secure *recognition* of the verbalized action by drawing on shared knowledge of a conventional iconic depiction of it.

In the next part of his response (line 38), Tomasz informs Ivar where he has put the rods. While naming the action a second time, he makes a two-stroke gesture by moving his left stretched arm with a flat hand open forward and slightly downward (Figure B). This is also a conventional gesture used for placing something somewhere. However, here the depiction simultaneously provides information about where the rods have been placed (cf. Kendon, 2004 on location-establishing actions). Tomasz moves his arm in the direction of the place and verbally refers to it with the locative adverb *der* (‘there’). In other words, his gesture combines action depiction with deictic reference as one meaningful package. The generic meaning of his arm movement is enriched by a specification of the location relative to Tomasz’s and Ivar’s position in the spatio-material configuration. Put differently, Tomasz’s specifying gesture is ‘environmentally coupled’ (Goodwin, 2007, 2018), as it incorporates elements of the surroundings and requires mutual orientation to the material space in order to make sense of it (cf. Streeck, 2008). This indicates that rather than merely securing the recognition of action, the employment of a specifying gesture serves to ensure the *understanding* of action performance by tying it to the material environment and exploiting the latter’s resources.

The selection of one mode of action depiction rather than another is clearly motivated locally and has several consequences. We have already shown that generic depictions highlight the relevance of the action itself while contextualized depictions mark the relevance of the contextual details of action performance. Each mode uses its own interpretative space which requires different orientations from both the speaker and the recipient. While generic depictions are based on a common conceptual space (common ground), their specifying counterparts refer to the contextually specific material space. Consequently, depending on the mode of action depiction, joint attention is constituted through different means. When selecting generic depictions, speakers mobilize resources that draw on assumed shared knowledge of conventional iconic aspects of the verbalized action. When producing contextualized depictions, they additionally orient to the material environment and attend to its relevant details. In this way, the recipient’s attention to the performance of generic gestures seems less important. This can be seen in Figure A from Extract 2: When Tomasz depicts the action, Ivar does not directly attend to his gesture but looks somewhere else. In the case of specifying gestures, the recipient’s attendance to the depiction seems necessary for the referred action to be properly understood (this principle cannot be shown in the above fragment but will be demonstrated in Extract 7).

### The Position of Action-Depicting Gestures

**FIGURE 3:**
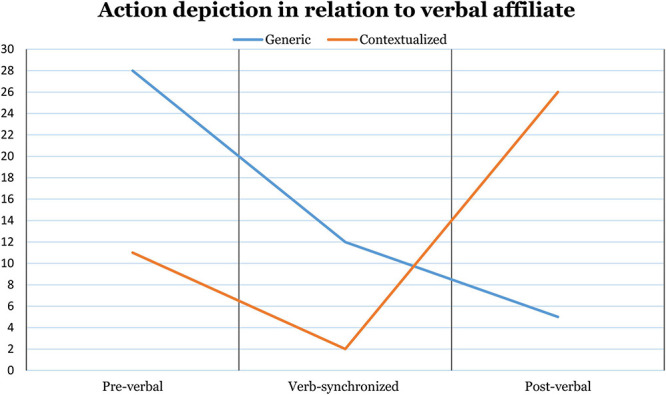
Change in the absolute frequency of the two modes of action depiction depending on their temporal positions relative to verbal affiliates (*n* = 84). Gestures with cross-extending strokes (*n* = 8), which begin pre-verbally and end post-verbally, are excluded.

Depending on the mode of action depiction, gesture strokes tend to occupy different positions in relation to their verbal affiliates. Generic depictions most often begin before the affiliate is verbalized (cf. *rydde* ‘clean up’ in the second turn construction unit in line 34, Extract 2), but they also make up the majority of the verb-synchronized gestures. Contextualized gestures, on the other hand, tend to occupy post-verbal positions, their strokes usually beginning when the verb is being verbalized and extending over the verb phrase or even beyond the turn (cf. line 38 in Extract 2). Figure 3 shows how the absolute frequency of specifying and non-specifying gestures in our data changes depending on their positions relative to the verbal affiliates.

In the following sections, we explore this positional variation with regard to grammatical and interactional environments. We describe each mode of action depiction in its standard and non-standard position, examining the local conditions of its employment.

### Generic Depictions in Pre-verbal Position

As we have demonstrated in the previous section, generic depictions have a strong tendency to occupy the pre-verbal and verb-synchronized position. Among the 48 identified non-specifying gestures, in 31 (65%) the stroke begins before the verbal affiliate while in 12 (25%) it is produced during the verbalization of the action verb. These gestures make up 66% and 86% of all gestures in each group, respectively. This raises a question about the reason and the meaning of such positioning in conversational interaction.

Embodied actions that recurrently precede units in talk are frequently used to foreshadow the occurrence of these units and thus to make them expectable (Streeck, 1995; Auer, 2005). This projective potential can be traced back through a systematic, retrospective observation of their occurrence relative to what comes after. Gestures have been demonstrated to project components or sequences of talk (Schegloff, 1984; Streeck, 1995), turn or sequence completion (Streeck, 1995; Mondada, 2006) or the next social action in conversation (Streeck, 1995, 2009b; Lilja and Piirainen-Marsh, 2019). Pre-positioned gestures have been considered to mark the projection space in which a lexical affiliate is expectable (Schegloff, 1984) and prefigure elements in talk that are relevant in the course of events (Streeck, 1995). Pre-verbal strokes in our data clearly reveal similar characteristics. Gestures in this position by default project the verbalizations of the relevant actions that receive prominence in the course of the turn-in-progress. This was seen in Extract 2 (line 34), where the pre-verbal depiction highlighted the salience of a new type of action (“cleaning up”) relative to action that was referred to in the question (moving the rods). The function of prepositioning is even more evident in Extract 3, where pre-verbal depiction is employed twice. Here Tomasz and Jonas (another Swedish superior who speaks Swedish-Norwegian) are talking about dismantling a formwork that is used to mold and hold fluid concrete in place until it hardens. When Tomasz remarks that there is a lot of concrete in the formwork (line 01), Jonas adds that they have winter conditions (line 03), implying that the drying process may take a long time. As a solution to this problem, he suggests turning on heating in order to speed up the drying (line 05).

**EXTRACT 3:**
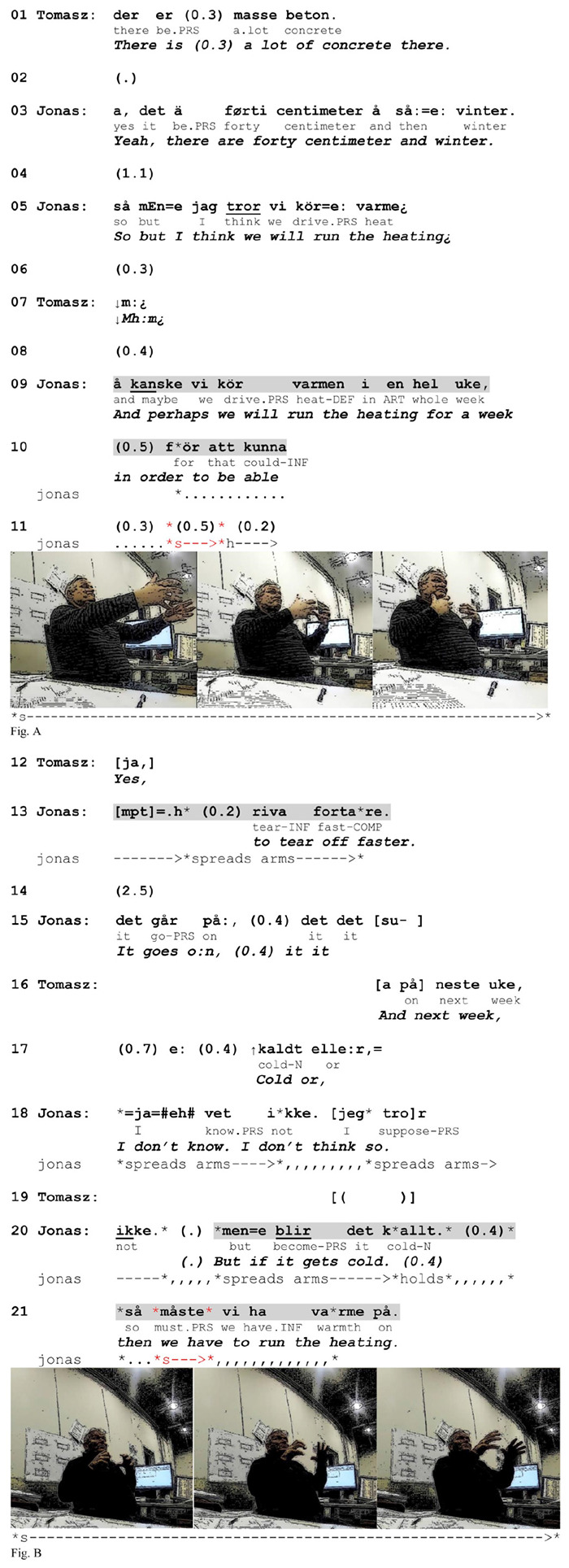
Swedish-Norwegian, office.

When Jonas suggests that they can run the heating for a whole week, he initiates an adverbial adjunct projecting an account for why they should do it (line 10: “in order to be able to”). However, before coming to the action-denoting verb, he aborts the utterance in course, and in the ensuing inter-turn pause he produces a one-stroke gesture depicting the action of tearing something off (Figure A). More specifically, in the preparation phase, he stretches both arms forward with his hands positioned vertically and his fingers slightly bent and loosened. In the stroke phase he moves his arms quickly inward, depicting the action of wrenching something off. This constitutes a generic depiction in that it does not display or refer to any features of the physical surroundings. Tomasz responds to the enactment with a minimal response that claims recognition of the depicted action (line 12) that Jonas then verbalizes and completes the clausal unit.

After this, Tomasz inquires whether it will be cold next week (lines 16–17). After initially producing a hedged negative response, Jonas initiates a conditional construction addressing the contingency that it might be (line 20). While producing the second part of the conditional, Jonas makes a one-stroke gesture by rapidly moving both arms forward and slightly opening his palms, depicting heat beaming from a heater (Figure B). This gesture is also produced before the verbal affiliate *ha varme på* (‘run the heating’) and does not involve contextual specifications.

**FIGURE 4:**
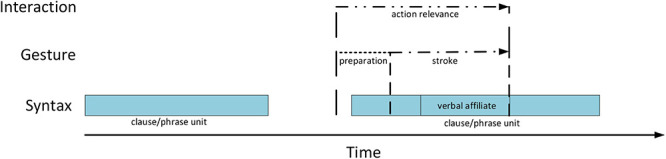
Temporal unfolding of generic pre-verbal gestures in their interactional and syntactic environment.

Both gestures from the above sequence display typical properties of generic depictions in pre-verbal position in the data. First, as in over 90% of the cases, the stroke is produced just before the verbal affiliate but after the initiation of the clause or phrase that serves as a syntactic matrix for the affiliate. In other words, the depicted action takes part in a larger syntactic projection. The trajectory of this projection in the three languages used in our data allows the recipient to anticipate the verbalization of an action soon after the stroke onset because the latter occurs right before the position where the syntactic frame requires a verb with the core semantic content (see Figure 4).^2^ Thus, pre-verbal gestures pre-introduce particular actions by drawing attention to what is coming at the level of verbalization, that is an action verb or a phrase that names the referred action.

Second, the gestures are typical of pre-positioned gestures in that they are used to draw the recipient’s attention to an upcoming action that the speaker indicates as salient for what he conveys at that point in the conversation^3^. It is worth noting that not all verbalized actions receive gestural depiction. In line 09 in Extract 3, the phrase *kör varmen* (lit. ‘drive warmth’) does not receive gestural representation but in line 21 a similar phrase does. These two fragments differ in terms of conversational topic. When Jonas produces the first depiction, he locally highlights that the performance of the referred action (‘tearing off’) is the aim that warrants the use of heating during a whole week. However, when he responds to Tomasz’s question about the weather, he highlights the action of using heating as warranted by the cold. Thus, in both cases the depicted action is made salient because it serves local purposes – the bodily enactment refers to the role of action performance (either warranting or warranted) in the course of events. As a result, prepositioned gestures prefigure the relevance of the upcoming action by representing it as locally salient for what the speaker is doing in interaction. The focus on the action itself excludes the relevance of any context-specific features and allows the speaker to rely on shared knowledge of depiction conventions.

This leads us to a conclusion that at least partly explains why non-specifying depictions most often occupy the pre-verbal position. It seems that their generic character speaks in favor of their employment right before the verbal affiliate and within the above mentioned syntactic and interactional constraints. Because an action, not its contextual specifics, is interactionally relevant, the speaker makes use of the conceptual space and draws on the generic representations of action performance. This seems to facilitate launching the gesture stroke earlier and marking that the action is in play, which, in turn, helps the recipient identify this action in advance. One reason is that generic depictions are easier and faster to design than specifying gestures. In the case of the latter, a motor scheme of action depiction must be adapted to the spatio-material environment and reference to this environment by default requires attracting the recipients’ attention to the gesture in order to ensure their orientation in the environment and thus their understanding of the specifics of the referred action. As far as generic depictions are concerned, attention to the specifics of the gesture is not required because the following verbalization secures the recognition of the depicted action anyway. Yet, this is far from claiming that the gesture increases redundancy. Its occurrence clearly manifests that the speaker prepares the recipient for the upcoming mention of the action and indicates that this action is salient in the context of what is being said.

### The Temporal Manipulation of Pre-positioned Gestures

In preparing the interlocutor for the upcoming verbalization of an action, speakers may use a range of spatio-temporal practices to render pre-verbal gestures more salient and recognizable. For instance, they may increase the length of the gestures or make them take up a large part of the gesture space. They may also position them in intra-turn pauses or complete the stroke before the onset of the affiliate. For instance, the first gesture in Extract 3 (line 11) above is produced in an extended intra-turn pause and with large arm movements, from maximal extension to maximal retraction of the hands. This increases its noticeability or witnessability (cf. Goodwin, 1986). Such an early completed stroke provides a possibility for the recipient to pay attention to the depiction and identify the referred action well before it is verbalized. Consequently, the temporal manipulation of pre-verbal gestures may serve as a pre-emptive practice in contexts where mutual understanding is at risk.

As we saw in that extract, Tomasz marks his understanding of the depiction before Jonas verbalizes the corresponding action (line 12 and 13). This instance illustrates a regularity observed in our data: The larger time gap between a gesture and its verbal affiliate is, the more witnessable the gesture becomes. Its witnessability, in turn, increases the chances of its recognition. In this way, non-specifying gestures in pre-verbal position establish their own relevance (cf. Streeck, 1995) and autonomy with regard to the talk, meaning that 1) their semantic content is independent of spatio-material constraints, and 2) in certain configurations they can work well without their linguistic affiliates, carrying the potential to co-constitute *syntactic-bodily units* (Keevallik, 2013). However, since our study focuses on the positional variation of action-depicting gestures relative to verbal affiliates, all pre-verbally represented actions that we have collected are additionally verbalized.

This raises a question about the role of action verbalization after the depiction has been completed and marked as recognized. In the example above, the verbalization can be seen as motivated by the fact that the speaker adds a temporal specification (the adverb *fortare* ‘faster’) and thereby needs to produce the main verb due to syntactic constraints. However, in Extract 4 there is no explicit evidence of such a motivation. Here Ivar explains to Tomasz when they will slow down work pace. When the sequence begins, Ivar points at a printed holiday schedule, marking a period he refers to with the adverb *här* ‘here’.

**EXTRACT 4:**
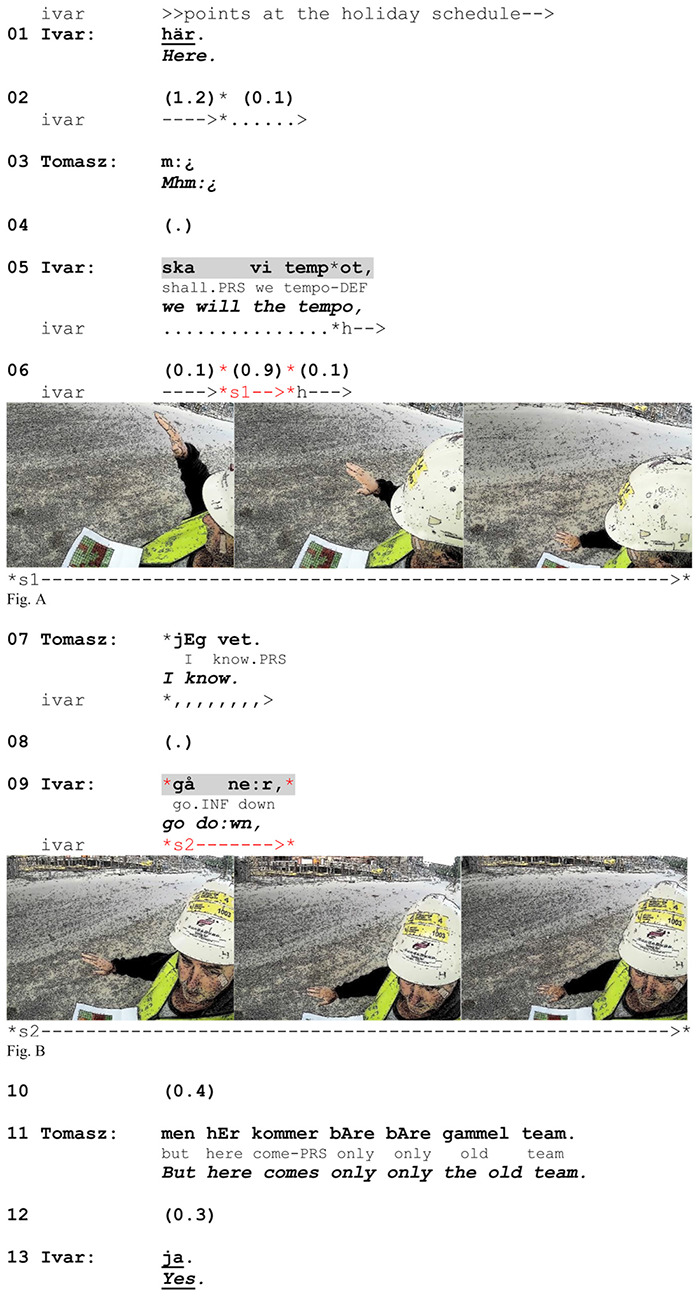
Swedish-Norwegian, site.

This adverb initiates a clausal unit that Ivar builds up incrementally. Before forming the second part of this unit (line 05), he prepares for the stroke by raising his right arm with the hand palm open down^4^. Then, as he suspends the delivery of the rest of the clause, he produces the gesture stroke by moving his arm downward (line 06, Figure A). The gesture is rendered particularly salient by a rather slow arm movement, making the gesture last for 0.9 s, and by a large excursion of the arm, from a very high to a very low position in the gesture space. Tomasz responds to his depiction with the strong epistemic assertion ‘I know’. By selecting this response form, he not only marks recognition of the referred action but also claims having prior access to the information conveyed (cf. Heritage and Sefi, 1992). Such a response has been characterized as resisting the informative value of the news (Sidnell, 2012) and even treating the delivered information as unnecessary (Mikesell et al., 2017). It is therefore surprising that Ivar subsequently decides to both verbalize the action in question and repeat the gesture, this time synchronized with the verbalization of the particle verb (line 09, Figure B). Relative to the first gesture, this one is enacted less prominently, taking up half as much time and using much less of the gesture space. One explanation for this seemingly redundant completion can be that he orients to Tomasz’s status as an L2 speaker and seeks to avoid the possibility of a misunderstanding. We have already seen that Ivar performs the first gesture in a rather exaggerated way and thereby seems to make extra efforts to secure recognition of the referred action. Furthermore, as Tomasz starts responding (line 07), Ivar starts turning his head toward him, thereby preparing to monitor him visually for evidence of understanding. The verbalization of action combined with the gesture and gaze directed at Tomasz (Figure B) thus reveals Ivar’s uncertainty about whether his previous multimodal utterance was indeed understood and seems to secure the recognition of the verbalized action one more time. After this, Tomasz does not respond to the delayed completion but instead changes the topic and proceeds with the activity in course (line 11). This indicates that he indeed treats the verbalization as redundant. We would argue that Ivar’s highly explicit form of expression, which would otherwise seem exaggerated and redundant, constitutes a pre-emptive practice oriented to securing mutual understanding in the face of potential problems related to limited linguistic resources in common.

### Synchronization of Gesture and Affiliate

The speakers adjust the gesture structure and the temporal unfolding of the stroke to the position of the verbal affiliate. It is a general feature of depictions that they may be dynamically adjusted to the temporal and syntactic conditions, and thus strokes can be multiplied if needed. However, 83% of non-specifying gestures in our data do not cross the right boundary of affiliate verbalization. In Extract 4, Ivar’s depiction consists of two strokes that compose the whole stroke phase. The second stroke is produced simultaneously with the verbalization. It is twice as short in time as the first one and ends with the end of the particle verb. This demonstrates that the verbal affiliate, naturally enough, is a pivot relative to which speakers most often organize their depictions temporally. Interestingly, for non-specifying enactments the affiliate usually marks out the last moment of depiction, while for the specifying ones it functions as the point of departure (see the sections below).

The second stroke of Ivar’s gesture is produced as a repetition of the enactment. Stroke synchronization with the verbal affiliate is found in repeated strokes in our data. It happens within a complex stroke phase in which one of the strokes aligns with the verb (as in Extract 4, Figure B) or independently, as a separate depiction aligned with a repeated affiliate. The example above gives us some clues as to how to understand the difference between the pre-verbal and the verb-synchronized positioning of action-depicting gestures. Ivar’s first gesture marks that what is structurally following the word *tempot* (‘tempo’) is an action of a certain type. However, when he repeats the same action while verbalizing it, he clarifies what action the gesture represents. This shows us two slightly different orientations toward the referred action, namely one that displays that the gesture is about an action and one demonstrating what action the gesture is about.

12 out of 14 gestures in our data that are independently synchronized with their verbal affiliates belong to generic depictions. Verb-synchronized gestures differ from the pre-verbal ones in some respect. On average, the stroke phase in the former is shorter and lasts 296 ms (median=300, SD=100) while in the latter it is 463 ms long (median=370, SD=373). Synchronization shows that speakers attend to the exact timing and the proper temporal extension of the stroke phase so that the enactment is produced within the boundaries of the verb production. Since this must be done in such a short time, the composition of a gesture is normally limited to a single stroke (all cases but one in the data).

Apart from repeated enactments, verb-synchronized gestures occur in two other environments in our data: They either depict one of two consecutive actions that is assumed by the speaker to be epistemically shared or represent an action the speaker has inferred from the prior talk and needs confirmation of. In Extract 5 below, we exemplify the use of a verb-synchronized gesture depicting an action that is inferred from talk. It shows how the gesture is used to support the naming of the action that seeks confirmation from the recipient. The sequence is a part of a conversation on the construction site in which Andreas tells Tomasz that the provisional wooden guardrails on one of the buildings can be dismantled. He does not name the action explicitly, but merely implies it by referring to what they ‘began yesterday’. While mentioning the particular elements he is referring to, Andreas points at them (lines 01–02). After this, Tomasz initiates two repair sequences in the form of understanding checks. First, he seeks confirmation of the intended referent by repeating the demonstrative pronoun with rising intonation and pointing at and moving his arm along the construction (line 04). Subsequently, he requests confirmation about the action to be taken by naming it with rising intonation (line 07) and depicting it with his right arm.

**EXTRACT 5:**
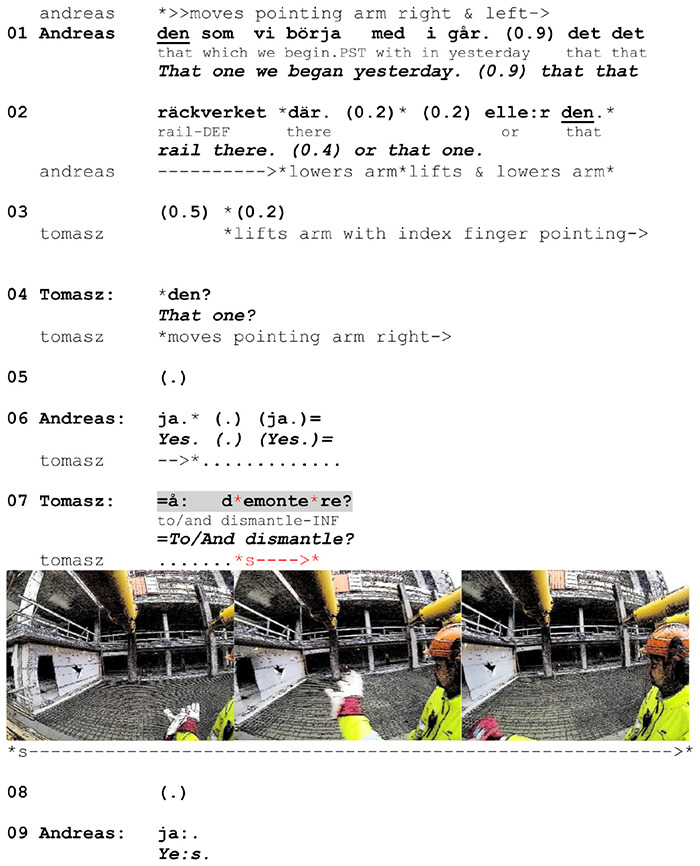
L2 Norwegian, site.

The stroke phase occurs within the boundary of the verbalized action verb and consists of one rapid arm movement from right to left that makes an arch-shaped trajectory. The gesture can be interpreted as an iconic depiction of decomposing or demolishing something. It does not provide any context-specific features of the referred action, such as directional or referential information. While producing it, Tomasz’s arm and fingers are not facing the construction (which might otherwise suggest that he simultaneously makes reference to it) but are positioned parallel to it.

As Andreas is not explicit about the action to be taken, Tomasz checks his understanding of Andreas’s utterance by explicitly naming the action and simultaneously depicting it in front of Andreas in a way that guarantees its visibility. Despite the fact that Andreas does not directly attend to the gesture, the depiction provides an additional cue that facilitates assessing whether the inference is right and responding to the question. The use of a synchronized gesture in the environment of a confirmation-seeking question additionally illustrates Tomasz’s inferential understanding. Here the role of the generic depiction is not to prefigure an upcoming action (as in the case of pre-verbal gestures) but to represent the particular action being expressed. As in gesture repetitions or descriptions of consecutive actions (where one action leads to another), this serves to ensure that the exact instance of the relevant action is properly recognized.

The above example displays one more detail that reveals speakers’ orientation to the generic depiction of action relevance. Tomasz establishes reference to the guardrail independently, before checking his understanding about the action to be taken. This represents a pattern found in the data: When action relevance is to be highlighted, speakers tend to establish the necessary contextual specifics of action performance separately. This provides evidence that speakers do distinguish action depiction in generic terms from action depiction in specific terms. In our case, context-specific features of the depicted action are treated separately, as Tomasz’s question highlights the action itself, not its local details (see also Extract 4).

So far, we have shown that generic action depictions in their most common positions follow regular patterns within the interactional and syntactic organization of talk. The verbal affiliate is treated as a pivot in this organization and action depiction is temporally adjusted and manipulated in advance of action verbalization. We have also demonstrated that when speakers synchronize gestures with their verbal affiliates, they distinguish between the relevance of action type and the relevance of a particular action. They also separate action depiction when it is to be understood in generic terms from its context-specific aspects if these need be indicated.

### Generic Depictions Extended Post-verbally

Five non-specifying gestures in our data are non-typical in that they occupy the post-verbal position, their stroke beginning when the verbalization of the referred action comes to an end. Another one starts before the verbalization and ends after turn completion. All these instances have two common features. First, the strokes are extended in time and cross the syntactic boundary of the turn, entering into turn transition space. Second, they are reacted to with (minimal) responses, alternatively, the verb and the gesture are reformulated until a response is produced. Consequently, we see this non-canonical use of generic depictions as a practice used for generating response from the interlocutor.

In Extract 6 we show how a non-specifying post-verbal gesture is used in a directive produced by a Swedish superior (Ivar) to the Polish worker (Tomasz). The gesture consists of two strokes. The first one begins while the verb is being verbalized and extends beyond it, ending in the turn transition space. The second one follows the former in the pause and ends while Tomasz is providing his acceptance of the directive.

**EXTRACT 6:**
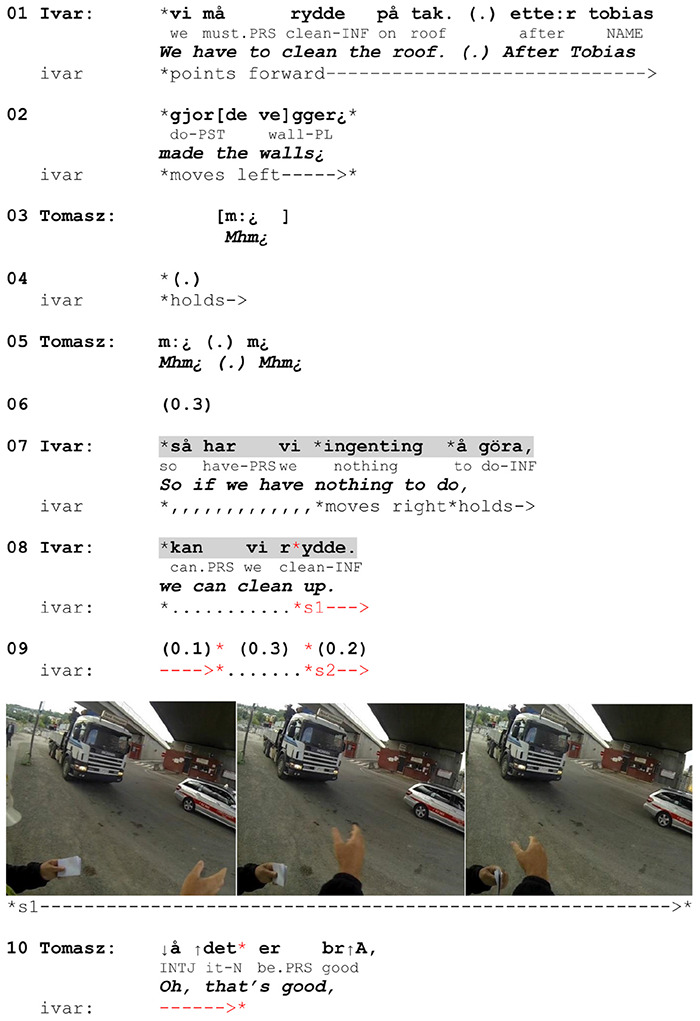
Swedish-Norwegian, site.

When Ivar says that the roof must be cleaned, he does not depict the verbalized action but instead points to where the cleaning is to be performed, thereby specifying the verbal place reference (line 01). In line 07, he elaborates the request by specifying that it is something they can do when there are no other pressing tasks. As he verbalizes the action again, he adds a gestural depiction by moving his right arm twice from right to left with the hand palm open vertically. This is a generic depiction of cleaning up (as also seen in Extract 2) and does not include deictic references to the roof or iconic depictions of the manner of action. The temporal span goes beyond the turn, prolonging the relevance of the referred action. The temporal orientation is clearly different compared to the previous ones: The depiction does not foreshadow an upcoming action (probably because it has already been named in line 01) but marks the relevance of responding. Tomasz’s reaction is delayed, following Ivar’s initiation of the second stroke long after the end of his turn. This provides evidence that the prolonged depiction may function as a device prompting response and that Tomasz orients to it as an indication that his reaction is anticipated. Importantly, Tomasz does not produce merely a minimal response but a construction he conventionally uses to mark acceptance, which reveals his orientation to the directive meaning of Ivar’s turn. Thus, the gesture co-participates in establishing mutual understanding in two ways. As a generic depiction it highlights the relevance of the action type the directive is built upon and indicates. As a post-verbally extended gesture it signals that the confirmation of uptake is expected.

Gestures crossing the boundaries of turns have been identified as a resource for signaling the social action of an utterance, making it intelligible for the recipient and thus establishing mutual understanding (Lilja and Piirainen-Marsh, 2019). And Kendon (1995) notes that the prolongation of gesture is a way of displaying that reply is expected. This is also observable in the extract below and the other five examples in our data. Speakers seem to extend the span of action depiction until they receive confirmation of uptake from their recipients. If response is lacking, they may reformulate the turn together with the gesture (which happens in one case in our data).

This leads us to the conclusion that these six cases represent a variant use of post-verbal strokes that contribute to the achievement of mutual understanding of the social action an utterance performs. By highlighting the relevance of action type, generic depictions facilitate and secure the recognition and visibility of a particular action. The identification of this action is crucial for understanding of the utterance’s function, which may explain why gesture prolongation in our data is used in instructions and directives (cf. Lilja and Piirainen-Marsh, 2019). In order to receive confirmation of mutual understanding, speakers extend depictions beyond the turn. We argue that such temporal extension makes non-specifying gestures marked, as they occupy a non-standard position relative to the affiliate. In this way they can draw recipients’ attention to the relevance of responding.

### Contextualized Depictions in Post-verbal Position

In 26 out of 39 (66%) specifying gestures in our data, the stroke occupies the post-verbal position, while in another 5 it begins pre-verbally but extends beyond the verbal affiliate. Contextualized depictions make up 79.5% of all gestures that cross the boundary of the affiliate and 84% of all strokes initiated during its verbalization at the earliest and completed post-verbally. In Extract 2 we have shown that the specification of action draws on the spatio-material environment and may be supported by deictic or directional components in talk, usually locative and directional adverbs. This is the case in slightly more than half the instances in our data. The other half does not include verbal indications of placement or direction. These depictions either mark the direction of action by means of gestures alone, or they focus on the *manner* of action performance. Extract 7 presents a case where a directional specification of depiction is not supported linguistically but relies on the recipient’s orientation to the gesture *in statu nascendi*.

**EXTRACT 7:**
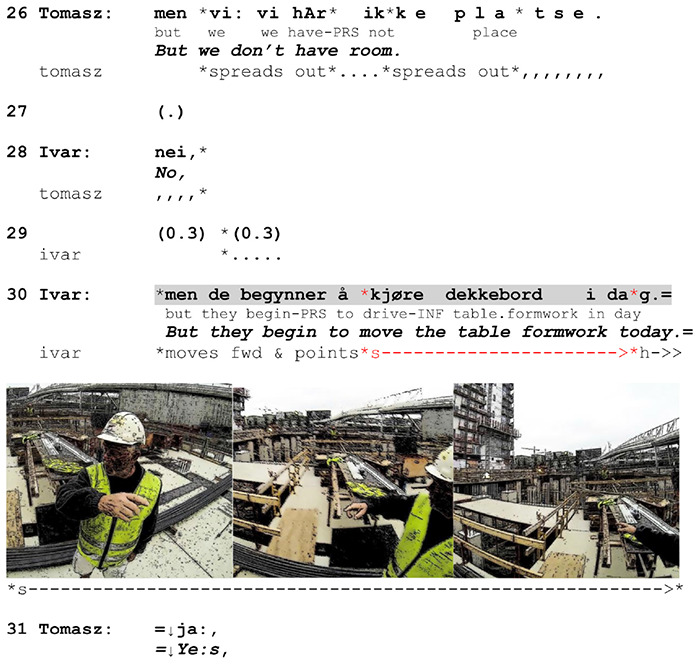
Swedish-Norwegian, site.

After Tomasz points out that they do not have enough space to perform the task he and Ivar have been talking about, Ivar notes that a table formwork will be moved that day, implying that this will solve the problem. Just as Ivar initiates his turn, he points with his right index finger to the left, making reference to one of the teams. Then he makes a long movement with his arm from left to right and stretches it at the end with the index finger pointing toward the other side of the construction site. Tomasz follows the enactment and manifests his understanding with a third-position receipt (line 31). The gesture illustrates an action of moving from one place to another. There are two details that speaks in favor of its depictive character: 1) the stroke starts with the verbalization of the action verb *kjøre* (‘drive’) and ends right before the turn ending, followed by a gesture hold; 2) the pointing is not a single and independent gesture but a part of a long, continuous movement. The depiction incorporates a concrete point of departure in the spatio-material environment, marks the trajectory and direction of the formwork movement, and makes deictic reference to the destination. By including these specifics in the embodied action, Ivar substantiates his implication that there will be more space. This supplementary information is crucial for understanding the contextual aspects of the verbalized action and thus the full meaning of Ivar’s response. The lack of locative adverbs in speech seems to be warranted by the fact that Tomasz is positioned opposite Ivar and attends to the gesture. Moreover, Ivar uses a verb *KJØRE* (‘drive’) that does not require a locative adjunct, and the context-specific details are not central to the point of his utterance, the main point being that the table formwork will be (re-)moved. This contrasts with Extract 2, where the verb *LEGGE* (‘lay’) required a locative adjunct and the central point of the utterance was to specify the contextual aspects, as the action itself had already been highlighted earlier in generic terms.

These two cases demonstrate how the combination of gestural and syntactic elements is motivated sequentially and used to accentuate and modulate the relevancies of action performance. Furthermore, we can also see that the supplementary use of different modalities (verbal and gestural) is a result of an interplay between syntactic constraints and interactional relevancies. Contextualized depictions can effectively represent indexical elements of action and thus be used instead of lexical items in cases where the latter are not syntactically required. But this effectiveness is achievable only when it is locally evident for the speaker that the recipient attends to the depiction.

Contextualized depictions in our data are employed more often on site than in the office (66% of the cases), which is not unexpected considering the fact that they draw on elements of the surrounding spatio-material environment^5^. The ones that do occur in the office are instead based on (pre-) establishment of proxy referents in the gesture space. In such cases, the organization of action depiction becomes more complex, as it must rely on available resources that can be used as substitutes: material (such as objects), abstract (such as graphic representations) and conceptual (shared knowledge on how certain things are done or on how certain objects are related to each other). In Extract 8, we demonstrate how such depictions are employed through a transposition of spatio-material arrangements from construction drawings to the gesture space. Jonas explains to Tomasz how they can block up a hole in a room in the construction site so that they can effectively use a heater in order to speed up concrete drying. Before naming the action, he points at a drawing on the table, clarifying which hole in the building he is referring to.

**EXTRACT 8:**
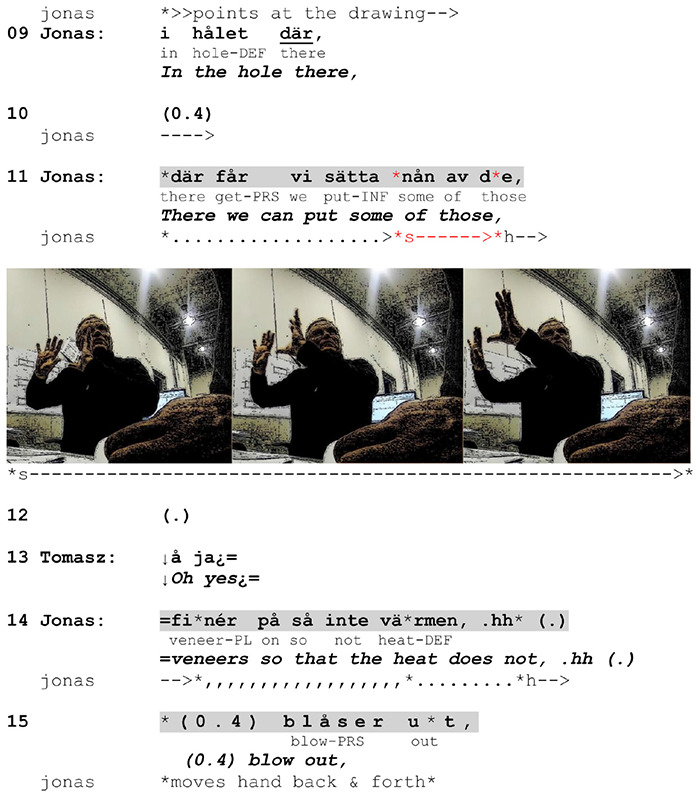
Swedish-Norwegian, office.

The drawing is an important point of departure for Jonas in his depiction of action. It provides information about spatial details that he incorporates to the gesture co-expressed with the next utterance (line 11) and makes Tomasz attend to. In the preparation phase, Jonas lifts both hands at the chest height and after producing the action verb (*sätta* ‘put’), he makes a stroke by moving them forward and slightly upward. The gesture depicts an action of putting some large, flat object with both hands at some height in order to cover the mentioned hole. Thus, the role of this enactment is not just to mark the relevance of putting something on something (which could be depicted with one hand) but to draw Tomasz’s attention to the specific *manner* of this action, namely how a veneer can be mounted in order to cover the hole. This involves marking a spatial relationship between hands and virtual objects in the gesture space. By securing understanding of these specifics, Jonas can secure understanding of his suggestion (cf. Lilja and Piirainen-Marsh, 2019). Importantly, Tomasz displays his recognition right after the stroke, in an intra-turn pause well before the utterance in course is complete. His change-of-state token *å ja* (‘oh yes’) compositionally and prosodically seems to treat the information as news. This indicates that Tomasz attends to the gesture, making sense of the combination of the verbalized action and its depiction.

### Contextualized Depictions in Pre-verbal Position

11 contextualized depictions in our data are positioned pre-verbally and do not cross the border of the verbal affiliate^6^. They make up 25% of all contextualized depictions and 23% of the gestures with strokes initiated pre-verbally. By means of their positioning, they prefigure both the verbal representation of the action and possible references to contextual aspects of its performance. This raises a question about the interactional motivations for this use of specifying gestures.

When analyzing these depictions in more detail, we find that they share two crucial features. The first is that, just as generic depictions in the same position, they occur within a larger syntactic projection that allows the recipient to anticipate the action. However, syntax does not necessarily project the verbalization of context-specific details of action performance. This can be observed in the previous example (Extract 8, line 15) where Jonas starts the depiction of ‘blowing out’ by referring with his hand to the drawing and marking where the air may escape. There is nothing in his turn that could indicate the place and the direction of action, yet he marks these specifics by positioning his hand and executing the stroke in a certain manner relative to the drawing.

Another feature that characterizes these gestures is that they are part of a larger gestural complex. In other words, before the stroke is produced, the relevance of gesture is already established and the recipient’s attention is secured by means of linguistic (e.g., deixis) and bodily (e.g., pointing, showing, walking) resources. This pattern was not observed in post-verbal specifying gestures, which are often used independently as single and momentary enactments. It seems therefore that the larger gesture complex supports and enables the depiction and recognition of context-specific elements of action performance before they are verbalized. In Extract 9, we show how gestural relevance is pre-established and how contextualized depiction relates to syntax. After finding out that the construction drawings lack information about additional stirrup reinforcement, Tomasz tells his Polish co-worker (Adam) about the problem. In lines 01–06 he first announces the problem and then introduces the solution that his Swedish superior had suggested earlier on.

**EXTRACT 9:**
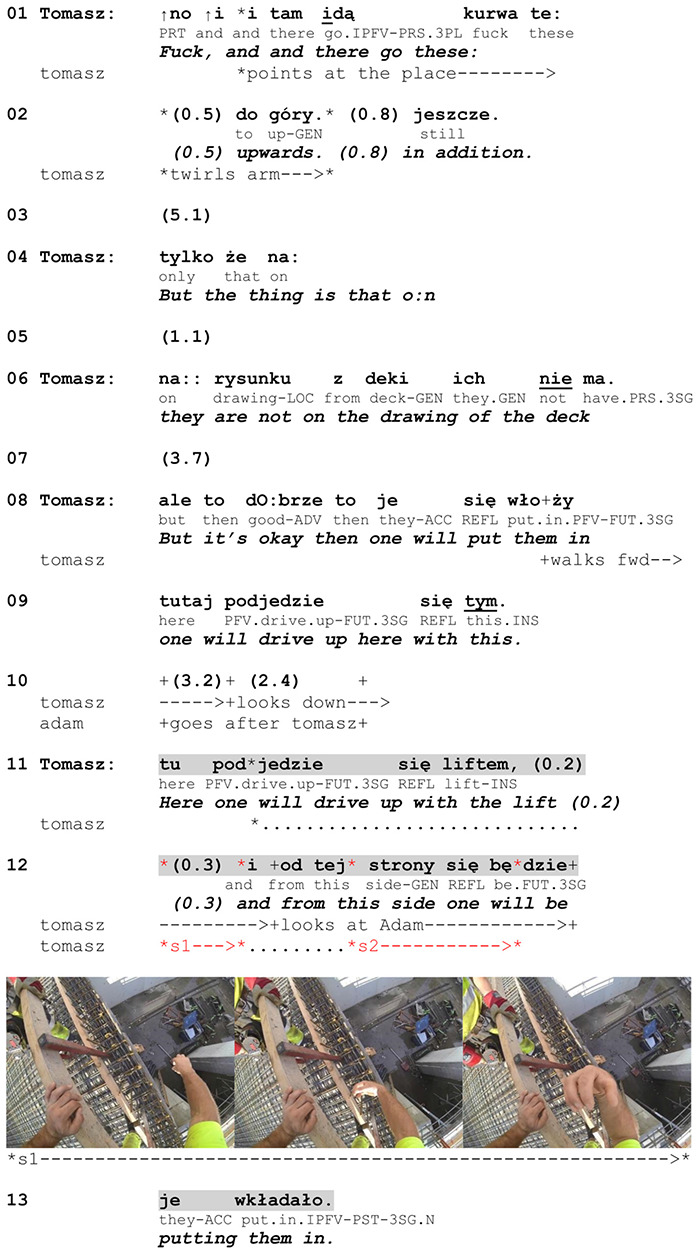
Polish, site.

When introducing the problem in lines 01–02, Tomasz uses the demonstrative *these* to refer to the stirrups and depicts the way in which they have to be anchored in the place he has just pointed at. After adding that this is the knowledge he had not had access to, he informs Adam about the solution and starts walking forward (line 08). The syntactic-bodily combination of the utterance *tutaj podjedzie się tym* (‘one will drive up here with this’) and Tomasz’s walking toward the relevant place is used to project a scene of action depiction. It also reveals that Tomasz does not treat his announcement of a solution as sufficient and clear enough for Adam. By walking to the place of action performance, he marks that he is going to show Adam more accurately how they can install the stirrups, thereby pre-establishing the relevance of a gestural depiction. At the same time, walking is also used to invite Adam to join him, which Adam accepts by following. Adam reaches the place a bit later than Tomasz, and Tomasz waits until Adam approaches him before he initiates the preparation phase by lowering his right arm (line 11). As a result, Adam’s attention to the upcoming depiction is established before the depiction is enacted.

Tomasz makes two similar strokes that illustrate how the stirrups can be installed on the reinforcing bars. Each consists of moving the arm upward with the wrist and the fingers bent inward. The first stroke comes in an inter-turn pause between two coordinated clauses. There are two local cues that indicate an action in progress. First, a fall-to-mid intonation contour of the first clause marks that a continuation is on the way. This modifies the semantic content of the clause, presenting the referred action (driving up with the lift) as a necessary precursor to some other action (in contrast to the clause in line 09). Second, right after the first stroke ends and before the second begins, Tomasz initiates the second clause with the conjunction ‘and’ followed by a topicalized adverbial constituent that highlights the manner and spatial dimension of action performance. The occurrence of the depiction before the topicalized components shows that it is first of all the contextual specifics of the action that the gesture projects. The topicalization of directional preposition phrase is one piece of evidence that the manner of action performance is given informational prominence. Moreover, the action has already been named (line 08) so in terms of topical focus, it is the manner of performance that becomes interactionally salient at this point^7^.

On the basis of the analysis of this extract, which is typical of this category of gestures, we argue that the pre-verbal positioning of contextualized depictions serves to draw the recipient’s attention to the upcoming specifics of action performance in cases where these aspects constitute the informational focus of the utterance. A necessary precondition for doing this is that the recipient’s attention to the gesture has already been secured. This explains the difference between the pre-verbal and the post-verbal positioning of contextualized depictions. While the latter serve to introduce context-specific details as an *addition* to an informational focus on the action itself, the former serve to make them salient as an independent focus of the utterance.

The example above again shows that the temporal position of action depiction is crucial in the way recipients orient to gesture content. Contextualized depictions in post-verbal position occur when the action is already verbalized, thus the stroke’s role is to carry additional information. In the pre-verbal position, the gesture marks the contextual specifics of action performance as informationally prominent.

## Discussion

The study has examined the positional variation of action-depicting gestures by focusing on the relationship between their semantic properties and their temporal unfolding relative to the verbal affiliates that named the depicted actions. We have specifically looked at why participants represent verbalized actions gesturally and how they execute this within syntactic units. By paying particular attention to the sequential organization of interaction, the study contributes to research on gesture and social interaction by showing that the employment and the temporal management of action-depicting gestures is motivated locally and their positional variation is meant to mark different pragmatic goals.

We have demonstrated that speakers use two modes of action depiction. The first one illustrates an action in generic terms, without referring to the elements of the spatio-material context (generic depiction). The second one represents an action in specific terms, adding to the depiction additional information about context-specific features of action performance as either complementary or supplementary content (contextualized depiction). Speakers use these modes to draw the recipients’ attention to different aspects of action performance that they want to make locally relevant. Generic depictions serve to highlight the relevance of action and facilitate its recognition by relying on shared knowledge of gestural conventions drawn from the conceptual space. Contextualized depictions are used to highlight the specifics of action performance (e.g., direction or manner) and facilitate its understanding by exploiting the material space. This distinction proposes an alternative view on the role of iconic gestures based on the situated significance of action features and the extent to which participants need to make use of the material context to generate their depictions. Our analysis provides empirical evidence revealing that speakers orient to these two modes of action depiction differently by manipulating its positional and temporal alignment with syntactic structures and by managing gestural composition in interactional turns in distinct ways.

Our study has demonstrated that the stroke of generic depictions is most often initiated before the verbalization of action or synchronized with the verbal affiliate while in the case of their contextualized counterparts it usually starts during the verbalization of action and extends beyond the verb phrase or the turn. We have argued that the earlier occurrence of non-specifying gestures is facilitated by the fact that generic depictions can be generated faster because they draw on already accessible knowledge assumed as shared. This is supported by local purposes, as the role of generic depictions is to draw attention to the action as a type, not to its specifics. Consequently, prepositioning foreshadows the salience of action type while synchronization highlights the particular action named in speech. Both serve to secure the recognition of the named action, either by providing cues as to what the recipient can expect as forthcoming or what exactly the speaker is indicating. Contextualized depictions, on the other hand, provide information based on a coupling of iconic illustration and referential specification within the spatio-material environment, which requires establishing the recipient’s attention to the gesture in order to make it understandable. This seems to take more time and interactional effort and explains the post-verbal positioning of these gestures. Through a depiction that is executed during and after action verbalization (the stroke sometimes being multiplied), the speaker can signal that s/he illustrates more than just the action that is already being represented verbally. In other words, it seems that the role of the post-positional expansion of action depiction is to make the recipient orient to the gesture as a means of providing additional information about the action.

The participants not only position their action-depicting gestures differently relative to the verbal affiliate but they also manipulate speech-gesture (a)synchrony. For instance, in order to pre-empt understanding problems and make the upcoming action more prominent, they may position the gesture in an intra-turn pause and complete it before the affiliate onset, which has the potential to attract the recipient’s attention. In our data almost half of the pre-verbal strokes were initiated during silence, and over 2 of 5 were completed before the affiliate. This challenges previous claims about the predominant stroke-speech synchronization (McNeill, 2005) and the reasons for potential asynchrony, such as speech disfluency or lexical retrieval difficulties (Krauss and Hadar, 1999; Chui, 2005). What we thus claim is that the temporal management of gestures is the resource that speakers use to mark different relevancies depending on their local needs and assessments. Word search might be one of them and trouble pre-emption another. This demonstrates that gesture-speech orchestration is a matter of flexibility, although limited. In interactional conditions, speakers adjust their embodied actions to the on-going talk and recipients make sense of this synergy by monitoring it. As we have shown, in some cases gestures provide sufficient information and the verbalization turns out to be redundant from the recipient’s point of view (cf. Schegloff, 1984). However, speakers generate their gestures (and recipients interpret them) by orienting to their temporal and structural constraints. These are marked out by syntax and the topical unfolding of interaction.

The dynamic temporal adjustment of action depiction happens in relation to syntactic components and units. In our data, the verbal affiliate is not only a semantic base that disambiguates the enactment but also a pivot of gestural alignment with speech. In most cases the verbalization of an affiliate was the moment of either gesture-speech synchronization, the completion of a pre-verbal depiction, or the beginning of a post-verbal one. This shows that the position of verbal constituents is consequential for speech-gesture coordination. Moreover, the temporal-syntactic span of speech before and after the affiliate provides an important orientation frame in the recognition of gesture. Pre-verbal strokes normally occur during an on-going syntactic projection that leads to the verbalization of action. Post-verbal strokes often align with syntactic slots where the referential specification of the depicted action normally happens in the three languages studied and to which speakers orient through stroke prolongation or multiplication. However, syntactic units and action-depicting gestures are parts of a larger interactional mechanism. Their production is steered by the local unfolding of talk and alterations in its topical composition. Speakers decide to depict actions that are salient to what they are conducting, either because a given action receives a special explanatory status in the course of events or because they want the recipient to understand the specifics of action performance. Furthermore, in the mechanics of turn-taking speech-gesture coordination is accompanied by other perceptual signals (Holler and Levinson, 2019). Additional gestures and other modalities, such as body movement, gaze, spatial ostentation etc., play an important role in distinguishing the different modes of action depiction and establishing the recipient’s attention. For instance, when an action is to be highlighted generically but refers to an object that needs referential specification, the latter is not incorporated into the depiction but provided by a separate deictic gesture.

**FIGURE 5:**
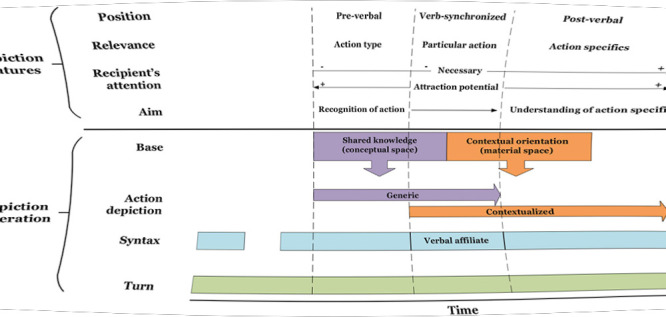
Temporal alignment of action-depicting gestures and speech in the turn.

The described patterns and mechanisms reveal their internal, systemic logic. The temporal alignment of action-depicting gestures with speech in interaction seems to have a fixed order. With the verbal affiliate as a pivot, the gestural occupation of its left side demonstrates clearly different characteristics of action depiction than the gestural occupation of its right side (Figure 5). Yet, this order is sometimes violated when specifying gestures occur before the affiliate or when non-specifying ones extend post-verbally. As we have shown, these deviations serve specific interactional purposes and are employed in certain conditions. Specifying gestures in pre-position served to informationally focalize the specifics of action but only when the recipient’s attention to the gesture was already established. Generic depictions in post-position, which extended beyond the turn, functioned to evoke the recipient’s manifestation of understanding, especially when this was crucial for the execution of the referred action.

Due to sample size, any reliable statistical analysis to support our claims was not possible. However, a detailed, systematic analysis of interactions has provided grounds for the generation of the hypothesis that generic action depictions are most often positioned before their verbal affiliates while their contextualized counterparts normally occupy the post-verbal position. Our study is a point of departure for future research to address the issue of gestural depiction and its temporal alignment with syntax by using larger samples of naturally occurring interactions in material environments and check the statistical power of our claims. Yet another interesting issue would be to investigate the positioning of action-depicting gestures in languages with different word order than SVO.

The last several decades of research on human interaction has shown that linguistic structures are only one of the resources we use to make and understand meanings as well as to organize our conversations. In everyday talk, participants smoothly combine language forms with embodied signals and the elements of the material world into meaningful multimodal packages or gestalts (Mondada, 2014). Recent advances in Conversation Analysis and psycholinguistics indicate that, as opposed to unimodal signals (e.g., merely linguistic), such packages may in fact facilitate predictive language processing by binding linguistic and gestural signals across multiple levels of expression (Holler and Levinson, 2019). Hand-arm-finger depictive gestures are just one of the modalities composing this multimodal complex. Yet, knowledge of how action depiction works allows us to learn about the details of intersubjective meaning making processes. This, in turn, provides valuable insight into the cognitive aspects of language use in its natural social environment.

## Data Availability Statement

The raw data supporting the conclusions of this article will be made available by the authors, without undue reservation.

## Ethics Statement

The studies involving human participants were reviewed and approved by Norwegian Centre for Research Data (NSD). The patients/participants provided their written informed consent to participate in this study. Written informed consent was obtained from the individual(s) for the publication of any potentially identifiable images or data included in this article.

## Author Contributions

Both authors were involved in the conception of the work and in the critical revision of the manuscript. PU: sample collection (gestures), transcription, coding, analysis, figures, tables, writing. JS: data collection, analysis, writing. Both authors contributed to the article and approved the submitted version.

## Conflict of Interest

The authors declare that the research was conducted in the absence of any commercial or financial relationships that could be construed as a potential conflict of interest.

## Publisher’s Note

All claims expressed in this article are solely those of the authors and do not necessarily represent those of their affiliated organizations, or those of the publisher, the editors and the reviewers. Any product that may be evaluated in this article, or claim that may be made by its manufacturer, is not guaranteed or endorsed by the publisher.

## Footnotes

1 Because Norwegian and Swedish are typologically close and mutually intelligible, we do not count the Swedish workers speaking Swedish-Norwegian as L2 speakers of Norwegian.

2 All three languages belong to the SVO type.

3 Tomasz’s response before the action verbalization (line 12) provides evidence that he attends to the depiction and recognizes the upcoming action.

4 Note that Ivar self-repairs in line 05 by changing the subject from ‘we’ to ‘the tempo’. It seems that this change is motivated by the early stroke preparation that starts in line 02. Because he is going to depict the action of ‘going down’, the change makes sense, as the phrase ‘the tempo will go down’ is semantically unequivocal and thus more understandable than the phrase ‘we will go down’.

5 In the case of non-specifying gesture this distinction does not provide any differences.

6 Another 2 contextualized depictions are synchronized with the verbal affiliate. One of them is a repetition of action, another one provides reference to a place to which the recipient’s spatial orientation is delayed. However, due to space limits, we decided to exclude this case from the analysis. Additional 5 instances of specifying gestures begin pre-verbally but end after the verbalization of the verbal affiliate (cross-extending strokes).

7 By the time Tomasz starts illustrating the action by showing how the stirrups will be installed, Adam has learnt about the object that the action refers to, why and where the action must be performed, what will be used to perform it, and what it is about.

## Appendix

**Table:**

**Transcription conventions**	
(1.1)	silence in seconds and tenths of a second
(.)	a micropause less than 0.2 s
↓↑	sharp changes in pitch
?	strongly rising intonation
¿	rising intonation
,	slightly rising intonation
.	falling intonation
[	beginning of overlapping talk
]	end of overlapping talk
ja=	latched talk
°ja°	quieter talk
ja::	sound stretching
j-	a cut-off
JA	louder talk or sound
ja	stress
.h	audible inhaling
(ja)	unclear/uncertain fragment
()	unintelligible talk
**Multimodal transcriptions**	
*	arm-hand-finger movement
+	other embodied actions (e.g., head movement)
*word*	two identical symbols delimit embodied actions
*s—->*	the exact moment of the beginning and the end of stroke
*h—->*	the exact moment of the beginning and the end of pre- or post-stroke hold
s1	stroke number in a stroke phase
……	action preparation
,,,,,,	action retraction
>>	the action begins before the excerpt’s or the turn’s beginning
*–>	the action continues across subsequent lines
–>*	the action ends at this point
–>06	the action ends in a line with the indicated number
–>>	the action continues after the excerpt’s end
**Morphological glosses**	
3	third person
ACC	accusative
ADV	adverb
ART	article
COMP	comparative
DEF	definite
FUT	future tense
GEN	genitive
INF	infinitive
INS	instrumental
IPFV	imperfective
LOC	locative
N	neuter
NAME	proper noun
PFV	perfective
PL	plural
PRS	present tense
PST	past tense
REFL	reflexive
SG	singular
